# The adverse effect of emergency department crowding on compliance with the resuscitation bundle in the management of severe sepsis and septic shock

**DOI:** 10.1186/cc13047

**Published:** 2013-10-06

**Authors:** Tae Gun Shin, Ik Joon Jo, Dae Jong Choi, Mun Ju Kang, Kyeongman Jeon, Gee Young Suh, Min Seob Sim, So Yeon Lim, Keun Jeong Song, Yeon Kwon Jeong

**Affiliations:** 1 Department of Emergency Medicine, Samsung Medical Center, Sungkyunkwan University School of Medicine, Seoul, Korea; 2 Department of Critical Care Medicine and Division of Pulmonary and Critical Care Medicine, Department of Medicine, Samsung Medical Center, Sungkyunkwan University School of Medicine, Seoul, Korea; 3 Department of Critical Care Medicine, Samsung Medical Center, Sungkyunkwan University School of Medicine, Seoul, Korea

## Abstract

**Introduction:**

The aim of this study is to evaluate the effects of emergency department (ED) crowding on the implementation of tasks in the early resuscitation bundle during acute care of patients with severe sepsis and septic shock, as recommended by the Surviving Sepsis Campaign guidelines.

**Methods:**

We analyzed the sepsis registry from August 2008 to March 2012 for patients presenting to an ED of a tertiary urban hospital and meeting the criteria for severe sepsis or septic shock. The ED occupancy rate, which was defined as the total number of patients in the ED divided by the total number of ED beds, was used for measuring the degree of ED crowding. It was categorized into three groups (low; intermediate; high crowding). The primary endpoint was the overall compliance with the entire resuscitation bundle.

**Results:**

A total of 770 patients were enrolled. Of the eligible patients, 276 patients were assigned to the low crowding group, 250 patients to the intermediate crowding group, and 244 patients to the high crowding group (ED occupancy rate: ≤ 115; 116–149; ≥ 150%). There was significant difference in compliance rates among the three groups (31.9% in the low crowding group, 24.4% in the intermediate crowding group, and 16.4% in the high crowding group, *P* < 0.001). In a multivariate model, the high crowding group had a significant association with lower compliance (adjusted odds ratio (OR), 0.44; 95% confidence interval (CI), 0.26 to 0.76; *P* = 0.003). When the ED occupancy rate was included as a continuous variable in the model, it had also a negative correlation with the overall compliance (OR of 10% increase of the ED occupancy rate, 0.90; 95% CI, 0.84 to 0.96, *P* = 0.002).

**Conclusions:**

ED crowding was significantly associated with lower compliance with the entire resuscitation bundle and decreased likelihood of the timely implementation of the bundle elements.

## Introduction

Severe sepsis and septic shock are life-threatening illnesses with a high mortality rate, whose incidence appears to be increasing in recent years [1-3]. Early identification and effective management in a timely fashion are key factors for improving survival in these patients [4-6]. Currently, the Surviving Sepsis Campaign (SSC) guidelines recommend implementing a resuscitation bundle to provide more rapid and qualified care. This bundle includes serum lactate measurement, early blood cultures and antibiotics, and early goal-directed therapy (EGDT) [4,7,8]. Compliance with the resuscitation bundle has been shown to reduce mortality, particularly when more components of the resuscitation bundle are accomplished within specific time limits [4,8-10].

Various barriers may interfere with the compliance and quality in the management of severe sepsis and septic shock [11,12]. In particular, emergency department (ED) crowding, which is one of the factors known to decrease the quality of the ED process, may have a potential effect on the care of sepsis patients [13,14]. Previous studies have shown that ED crowding delays appropriate care for several populations including the time required for administration of antibiotics for pneumonia patients and febrile neonates, for brain imaging in patients with acute stroke, for percutaneous coronary intervention for acute myocardial infarction, and for analgesia in adults with severe pain [15-19]. However, the association between ED crowding and compliance with the resuscitation bundle has not been determined.

The objective of this study was to evaluate the effects of ED crowding on the implementation of tasks in the early resuscitation bundle during acute care of patients with severe sepsis and septic shock. We hypothesized that the degree of ED crowding was associated with the rate of achievement of the resuscitation bundle.

## Methods

We analyzed the sepsis registry for patients presenting to the ED and meeting the criteria for severe sepsis or septic shock and data were prospectively collected from August 2008 to March 2012 at Samsung Medical Center (a 1,960 bed, university-affiliated, tertiary referral hospital with 70,000 annual ED visits in Seoul, South Korea). There are 58 beds for patients including hallway spaces in the ED of the study hospital (50 beds in the adult zone and eight beds for the pediatric zone). The registry was previously used in our studies regarding severe sepsis and septic shock [20-22].

The study was approved by the institutional review board of Samsung Medical Center. Informed consent was waived because of the retrospective observational and anonymous nature of the study.

### Patient inclusion criteria

We included patients, 18 years of age or older, who presented with septic shock or severe sepsis and blood lactate concentrations of ≥4 mmol/L. Exclusion criteria were as follows: (1) terminal malignancy, (2) patients who had previously signed ‘Do Not Resuscitate’ (DNAR) orders, and (3) patients who refused the EGDT. We excluded patients with terminal malignancy because these patients are not often provided with intensive treatment, and they instead usually receive only conservative treatment.

### Definition

Sepsis was defined as suspected infection in the presence of two or more systemic inflammatory response syndrome criteria [23]. Severe sepsis was defined as sepsis associated with acute organ dysfunction [24]. Septic shock was defined as persistent hypotension (systolic arterial pressure <90 mmHg, mean arterial pressure (MAP) <60 mmHg, or a reduction in systolic blood pressure >40 mmHg from baseline) despite adequate volume resuscitation [24]. Cryptic shock was defined as normotensive patients with blood lactate concentrations ≥4 mmol/L [25]. Initiation of EGDT was defined as central line insertion followed by measurement of central venous oxygen saturation (Scvo_2_) within six hours from the time of meeting the criteria for resuscitation [12].

### Resuscitation protocol and bundle

A protocol for early recognition and appropriate management of patients with severe sepsis or septic shock was provided for the ED physician, included in our previous study [22], and was based on the protocol by Rivers *et al*. [6]. We recommended delivery of the resuscitation bundle to hemodynamically stable sepsis patients with serum lactate levels ≥4 mmol/L or systolic BP <90 mmHg after initial volume resuscitation, based on the 2008 SSC guidelines [7]. Initial evaluation and resuscitation were conducted in the ED. When severe sepsis or septic shock was suspected, patients were assigned to a critical care zone in the ED, which included five beds, advanced monitoring equipment and mechanical ventilators.

The resuscitation bundle was categorized by seven interventions [4,8,10]: 1) serum lactate measurement; 2) blood culture before antibiotic administration; 3) broad spectrum antibiotics administered within three hours from the time of presentation in the event of hypotension and/or lactate ≥4 mmol/L; 4) delivery of an initial minimum volume of 20 ml/kg crystalloid (or colloid) in the event of persistent hypotension despite fluid resuscitation and/or lactate ≥4 mmol/L; 5) achievement and maintenance of MAP ≥65 mmHg; 6) achievement of central venous pressure (CVP) ≥8 mmHg; and 7) achievement of Scvo_2_ ≥70%.

‘Time zero’ was defined as the time at which criteria for initiation of the specific intervention were met [4,10]. For calculating the time for lactate measurement, blood cultures, and antibiotic administration, time zero was defined as the moment of presentation, which was considered as the time of triage [8]. Time zero to initiate the EGDT was considered when either hypotension or hyperlactatemia greater than 4 mmol/L was reported.

The bundle compliance was assessed as whether or not a particular task was completed [4,9,10]. Compliance with each single intervention was considered as having been achieved if it was implemented within six hours from time zero, except for antibiotic therapy. Results are shown as the number of bundle interventions completed (0 to 7).

### Emergency department crowding measurement

The ED occupancy rate, which was defined as the total number of patients in the ED divided by the total number of ED beds, was used for measuring the degree of ED crowding [26]. It is a simple and validated tool for real-time assessment of crowding, and it has been widely used in other studies [15-17,19,26,27]. We tallied the total number of patients in the specific zone of the ED every hour by using an electronic medical records system. The ED occupancy rate was calculated from the number of patients in the ED at the time of triage. The occupancy rate was computed based on the adult ED section, including the waiting zone. The ED occupancy rate was categorized into tertile groups as follows: low crowding group, intermediate crowding group, and high crowding group [27]. We also examined whether the beds of the critical care zone were at full capacity when sepsis patients arrived at the ED or when they were diagnosed with severe sepsis.

If the critical care zone is at full capacity, we try to transfer the patients, who are occupying critical zone, to ICU, operating theater or general ward within the same facility to recover the capacity for the critical care zone. As a regional tertiary referral center, the study hospital does not transfer critically ill patients, including those with severe sepsis, to other hospitals.

### Outcome measurement

The primary endpoint was the overall compliance of the entire resuscitation bundle. Secondary endpoints were the completion rate of each intervention, the number of bundle interventions completed, the ED length of stay (LOS), ICU LOS, in-hospital LOS, and in-hospital mortality.

### Data collection

Data were obtained from our sepsis registry and electronic medical records. Potential risk factors influencing compliance with the bundle were considered when we chose variables [12,21]. We collected patient demographics, site of infection, vital signs, and laboratory data. Sequential Organ Failure Assessment (SOFA) scores were calculated at the time of severe sepsis or septic shock diagnosis from the data obtained [28]. Poor performance status was defined as confined to bed 50% or more of waking hours. We recorded the mode of arrival (use of emergency medical service) and time variables, such as the time of presentation, time of hypotension or hyperlactatemia, and time when specific interventions were done. The time period was categorized according to the routine changes of the number of nurses and physicians. In addition, the physician’s gender and the level of experience (years in the field) possessed by the physicians and nurses were also recorded.

### Statistical analysis

Continuous variables were expressed as the median with interquartile ranges because the majority of the data did not follow a normal distribution. Categorical variables were expressed as numbers (percentages) of patients. Continuous variables were compared using the Kruskal-Wallis rank test or the Wilcoxon rank-sum test according to the number of groups. Additionally, we assessed trends in the compliance, the number of completed interventions, and the implementation timing across the crowding levels using the Wilcoxon-type test for trend analysis. Categorical variables were compared with the chi-square test or the Fisher’s exact test. For all multiple comparisons, *P*-values were calculated by applying a Bonferroni correction.

Unadjusted and adjusted odds ratios (ORs) for primary outcome measures were calculated by univariate and multivariate logistic regression analysis, respectively. Variables which were found to be statistically significant at *P* <0.10 using univariate analysis were selected and included in the final multivariate models. Several variables were mandatorily adjusted regardless of *P*-value, including age, gender, body temperature, cryptic shock, SOFA score, initial lactate level, the time period of ED arrival, physician’s gender, the level of experience of physicians and nurses, and the study period. First, we made a model for the overall compliance. The probability of overall compliance was calculated from the multivariate model. Subsequently, we conducted logistic analysis for compliance with each element in the same way. The Hosmer-Lemeshow test was used to check the goodness-of-fit of the logistic regression.

Stata 12.0 was used for statistical analysis, and a two-tailed *P* value <0.05 was considered significant.

## Results

### Baseline characteristics

We identified 917 patients with severe sepsis or septic shock during the study period. We excluded 116 patients with terminal malignancy, 27 DNAR patients, and four patients who refused EGDT. Finally, a total of 770 patients were included in this study. Of eligible patients, 276 patients were assigned to the low crowding group, 250 patients to the intermediate crowding group, and 244 patients to the high crowding group (ED occupancy rate: ≤115; 116 to 149; ≥150%). The median ED occupancy rate was 132% (interquartile rage, 110 to 162%).

Comparison of baseline characteristics among the three groups is summarized in Table 1. There were no significant differences except for the presence of chronic renal disease and the time period of ED arrival. Patients who presented during the nights or weekends were more common in the low and intermediate crowding groups.

**Table 1:** Comparison of baseline characteristics

	**Overall group**	**Low crowding group**	**Intermediate crowding group**	**High crowding group**	***P***
	**(number = 770)**	**(number = 276)**	**(number = 250)**	**(number = 244)**	
Age (years)	65 (55 to 73)	64 (53 to 72)	67 (56 to 73)	65 (54 to 74)	0.081
Gender (male)	437 (56.8)	161 (58.3)	131 (52.4)	145 (59.4)	0.232
Comorbidities					
Hypertension	244 (31.7)	76 (27.5)	82 (32.8)	86 (35.2)	0.152
Diabetes	165 (21.4)	52 (18.8)	56 (22.4)	57 (23.4)	0.411
Cardiovascular disease	79 (10.7)	26 (9.4)	27 (10.8)	26 (10.7)	0.847
Chronic lung disease	48 (6.2)	17 (6.2)	18 (7.2)	13 (5.3)	0.690
Chronic renal disease	35 (4.5)	4 (1.4)	22 (8.8)^a^	9 (3.7)	<0.001
Chronic hepatic disease	67 (8.7)	19 (6.9)	21 (8.4)	27 (11.1)	0.235
Metastatic solid cancer	194 (25.2)	65 (23.6)	58 (23.3)	71 (29.1)	0.241
Hematologic malignancy	99 (12.9)	36 (13.1)	29 (11.6)	34 (13.9)	0.734
Organ transplantation	14 (1.8)	6 (2.2)	3 (1.2)	5 (2.1)	0.667
Neutropenia (ANC <500 mm^3^)	131 (17.0)	48 (17.4)	42 (16.8)	41 (16.8)	0.978
Nursing home resident	29 (3.8)	9 (3.7)	9 (3.6)	11 (4.5)	0.741
Poor performance status	35 (4.6)	11(4.0)	12 (4.8)	12 (4.9)	0.854
Suspected infection focus					0.259
Intra-abdominal infection	288 (37.4)	112 (40.6)	92 (36.8)	84 (34.4)	
Pneumonia	218 (28.3)	77 (27.9)	71 (28.4)	70 (28.7)	
Urinary tract infection	115 (14.9)	40 (14.5)	44 (17.6)	31 (12.7)	
Others	149 (19.3)	47 (17.0)	43 (17.2)	59 (24.2)	
Initial vital signs					
Mean arterial pressure (mmHg)	66 (58 to 80)	66 (58 to 81)	65 (57 to 78)	67 (59 to 81)	0.185
Heart rate (per minute)	112 (95 to 130)	115 (96 to 135)	109 (94 to 126)	112 (95 to 130)	0.065
Respiratory rate (per minute)	20 (20 to 24)	20 (20 to 24)	20 (20 to 24)	20 (19 to 24)	0.271
Body temperature (°C)	38.0 (36.8 to 38.9)	38.3 (37.0 to 38.9)	37.9 (36.7 to 38.8)	37.9 (36.8 to 38.9)	0.165
Initial presentation of cryptic shock	288 (37.4)	103 (37.2)	95 (38.0)	90 (36.9)	0.967
Initial serum lactate (mmol/L)	4.3 (2.5 to 5.7)	4.4 (2.8 to 5.8)	4.4 (2.4 to 5.7)	4.2 (2.5 to 5.4)	0.531
SOFA score	7 (4 to 9)	7(4 to 10)	7(4 to 9)	7(4 to 9)	0.458
Mechanical ventilation	87 (11.3)	32 (11.6)	28 (11.2)	27 (11.1)	0.980
Time period of initial presentation					
Night (10 PM to 7 AM)	191 (24.8)	122 (44.2)	60 (24.0)^a^	9 (3.7)^ab^	< 0.001
Weekend	227 (29.5)	162 (58.7)	58 (23.2)^a^	7 (2.9)^ab^	< 0.001
Physician’s experience					0.217
≤ 2^nd ^year residents	602 (78.2)	212 (76.8)	190 (76.0)	200 (82.0)	
≥ 3^rd ^year residents or	168 (21.8)	64 (23.2)	60 (24.0)	44 (18.0)	
board-certified physicians					
Physician’s gender (male)	381 (49.5)	135 (48.9)	114 (45.6)	132 (54.1)	0.163
Nurse’s experience					0.115
0 to about 2 years	397 (51.6)	152 (55.3)	136 (54.4)	109 (44.7)	
3 to about 5 years	215 (28.0)	73 (26.6)	67 (26.8)	75 (30.7)	
More than 5 years	157 (20.4)	50 (18.2)	47 (18.8)	60 (24.6)	
Use of EMS	247 (32.2)	88 (32.1)	81 (32.4)	78 (32.1)	0.997
Study period					0.221
2008 to about 2009	291 (37.8)	104 (37.7)	104 (41.6)	83 (34.0)	
2010 to about 2012	479 (62.2)	172 (62.3)	146 (58.4)	161 (66.0)	
Critical care zone at full capacity	55 (7.1)	9 (3.3)	10 (4.0)	36 (14.6)	< 0.001
ED occupancy rate (%)	132 (110 to 162)	100 (92 to 111)	136 (126 to 142)	172 (163 to 187)	

Data are shown as median with interquartile range or number (%). ^a^*P *<0.05 compared with the low crowding group after Bonferroni correction; ^b^*P *<0.05 compared with the intermediate crowding group after Bonferroni correction. ANC, absolute neutrophil count; EMS, emergency medical service; SOFA, Sequential Organ Failure Assessment.

### Resuscitation bundle compliance

**Figure 1:**
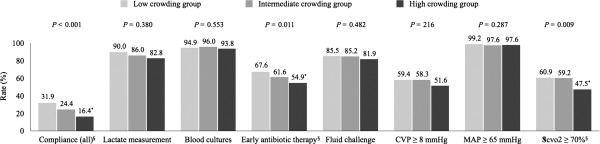
Compliance with the resuscitation bundle according to tertile of the ED occupancy rate; ^*^indicates *P *<0.05 compared with the low crowding group after Bonferroni correction; ^**§**^**indicates *P *for trend <0.05 among the tertile groups; CVP, central venous pressure; MAP, mean arterial pressure.** ED, emergency department.

Compliance with the entire resuscitation bundle was 25.6% in the study population. There was a significant difference in compliance rates between the three groups (31.9% in the low crowding group, 24.4% in the intermediate crowding group, and 16.4% in the high crowding group, *P* <0.001, *P* for trend = 0.007) (Figure 1). In particular, the absolute difference between the low group and the high crowding group was 15.5% (*P* <0.001). Among the elements of the resuscitation bundle, the completion rates of early administration of antibiotics and the achievement of Scvo_2_ ≥70% were significantly lower in the high crowding group than in the low crowding group (*P* = 0.009 and *P* = 0.006, respectively) and also showed significant decreasing trends.

The number of completed interventions in the bundle was significantly lower in the high crowding group compared with the low (*P* <0.001) and intermediate crowding groups (*P* = 0.013) (Table 2). When we analyzed the time intervals from time zero to the implementation of each bundle component according to tertile, there were consistently increasing trends toward the time intervals from time zero to lactate measurement (*P* = 0.007), blood cultures (*P* = 0.038), broad-spectrum antibiotic use (*P* = 0.005), fluid challenge (*P* = 0.036), the achievement of CVP ≥8 mmHg (*P* = 0.003), use of vasopressors (*P* = 0.004), and the achievement of Scvo_2_ ≥70% (*P* = 0.016).

**Table 2:** Number of completed interventions and the time intervals from time zero to the implementation of each bundle component

	**Overall group**	**Low crowding group**	**Intermediate crowding group**	**High crowding group**	***P***
	**(number** ***=*** **770)**	**(number = 276)**	**(number = 250)**	**(number = 244)**	
Number of interventions of the bundle accomplished^c^	5 (4 to 6)	6 (5 to 7)	6 (5 to 6)	5 (4 to 6)^ab^	<0.001
Time to the implementation^d^ (hours)					
Serum lactate measurement^c^	1.3 (0.8)	1.2 (0.8 to 2.5)	1.3 (0.9 to 3.0)	1.4 (0.9 to 3.6)	0.023
Blood cultures^c^	1.2 (0.8 to 2.1)	1.2 (0.8 to 1.9)	1.2 (0.8 to 2.3)	1.3 (0.9 to 2.2)	0.109
Broad-spectrum antibiotics^c^	2.5 (1.7 to 3.8)	2.4 (1.5 to 3.5)	2.6 (1.7 to 3.9)	2.8 (1.8 to 4.2)	0.018
Intravenous fluid challenge^c ^(n = 649)	0.6 (0.1 to 1.5)	0.6 (0.1 to 1.5)	0.6 (0.1 to 1.6)	0.8 (0.2 to 1.6)	0.109
CVP ≥8 mmHg achieved^c ^(n = 546)	2.9 (1.7 to 5.5)	2.5 (1.5 to 5.0)	2.7 (1.5 to 4.9)	3.5 (2.0 to 6.8)^ab^	0.005
Use of vasopressors^c ^(n = 473)	2.1 (1.1 to 3.4)	1.8 (1.0 to 3.1)	1.9 (1.1 to 3.1)	2.4 (1.4 to 4.2)^ab^	0.003
Scvo_2_ ≥70% achieved^c ^(n = 573)	3.5 (1.9 to 6.6)	3.3 (1.7 to 5.7)	3.5 (2.0 to 5.8)	4.2 (1.9 to 6.5)	0.051

Data are shown as median with interquartile range or number (%). ^a^*P *<0.05 compared with the low crowding group after Bonferroni correction; ^b^*P *<0.05 compared with the intermediate crowding group after Bonferroni correction; ^c^*P *for trend <0.05 among the tertile groups; ^d^calculated beyond the specific time limits. CVP, central venous pressure; MAP, mean arterial pressure.

### Logistic regression analysis

The uni- and multivariable analyses for the overall compliance are shown in Table 3. After adjusting for potential confounders, the high ED crowding was associated with lower sepsis care compliance (adjusted OR, 0.44; 95% confidence interval (CI), 0.26 to 0.76; *P* = 0.003) (Table 3). When we conducted a stratified analysis based on physician experience, which might have interactions with crowding, we found a significant correlation between crowding and compliance in all subgroups (adjusted OR, 0.50 (95% CI, 0.26 to 0.96; *P* = 0.037) in the subgroup of physician’s experience ≤2^nd^ year residents and adjusted OR, 0.25 (95% CI, 0.08 to 0.72; *P* = 0.010) in the subgroup of physician’s experience ≥3^rd^ year residents).

**Table 3:** Univariate and multivariate analyses for overall compliance with the entire resuscitation bundle

**Variable**	**Univariate**		**Multivariate**	
	**Unadjusted OR (95% CI)**	***P***	**Adjusted OR (95% CI)**	***P***
Tertile groups of crowding				
Low	Reference		Reference	
Intermediate	0.69 (0.46 to 1.01)	0.058	0.70 (0.45 to 1.09)	0.116
High	0.41 (0.27 to 0.64)	<0.001	0.44 (0.26 to 0.76)	0.003
Age >65 years	1.15 (0.83 to 1.61)	0.382	1.16 (0.82 to 1.66)	0.391
Female gender	1.02 (0.73 to 1.42)	0.901	1.15 (0.82 to 1.67)	0.429
Comorbidities				
Hypertension	1.14 (0.80 to 1.62)	0.460		
Diabetes	0.86 (0.57 to 1.30)	0.475		
Cardiovascular disease	0.90 (0.52 to 1.56)	0.701		
Chronic lung disease	0.60 (0.27 to 1.30)	0.193		
Chronic renal disease	1.07 (0.49 to 2.32)	0.869		
Chronic hepatic disease	0.64 (0.34 to 1.24)	0.190		
Metastatic solid cancer	0.81 (0.55 to 1.20)	0.295		
Hematologic malignancy	1.05 (0.65 to 1.71)	0.842		
Organ transplantation	0.51 (0.11 to 2.30)	0.381		
Neutropenia (ANC <500 mm^3^)	0.90 (0.57 to 1.40)	0.631		
Nursing home resident	0.79 (0.32 to 1.98)	0.621		
Poor performance status	0.62 (0.26 to 1.53)	0.302		
Suspected infection focus				
Others	Reference			
Intra-abdominal infection	0.98 (0.62 to 1.57)	0.962		
Pneumonia	0.86 (0.53 to 1.42)	0.559		
Urinary tract infection	1.48 (0.86 to 2.56)	0.150		
Heart rate >100 per minute	0.90 (0.63 to 1.27)	0.545		
Respiratory rate >24 per minute	1.03 (0.68 to 1.56)	0.886		
Body temperature >38.0°C	1.50 (1.07 to 2.09)	0.016	1.68 (1.17 to 2.40)	0.005
Initial presentation of cryptic shock	0.35 (0.24 to 0.52)	<0.001	0.39 (0.24 to 0.65)	<0.001
Lactate >4 mmol/L	0.55 (0.40 to 0.78)	0.001	0.80 (0.53 to 1.22)	0.311
SOFA score ≥8	1.93 (1.38 to 2.69)	<0.001	1.46 (1.00 to 2.13)	0.051
Mechanical ventilation	0.72 (0.41 to 1.25)	0.251		
Time period, night (10 PM to 7 AM)	1.34 (0.92 to 1.94)	0.116	0.93 (0.60 to 1.42)	0.725
Time period, weekend	1.47 (1.03 to 2.10)	0.032	1.24 (0.81 to 1.92)	0.313
Physician’s experience				
≤ 2^nd^ year residents	Reference		Reference	
≥ 3^rd^ year residents or	2.51 (1.74 to 3.63)	<0.001	2.85 (1.90 to 4.28)	<0.001
Board-certified physicians				
Physician’s gender (male)	0.93 (0.67 to 1.29)	0.673	0.83 (0.58 to 1.19)	0.318
Nurse’s experience				
0 to about 2 years	Reference		Reference	
3 to about5 years	1.38 (0.94 to 2.01)	0.093	1.56 (1.04 to 2.36)	0.030
More than 5 years	1.01 (0.65 to 1.58)	0.948	1.16 (0.73 to 1.88)	0.604
Use of EMS	1.12 (0.79-1.60)	0.496		
Study period				
2008 to about 2009	Reference		Reference	
2010 to about 2012	0.98 (0.70 to 1.37)	0.921	1.19 (0.82 to 1.73)	0.369
Critical care zone at full capacity	0.66 (0.33 to 1.35)	0.258		

ANC absolute neutrophil count; CI, confidence interval; EMS, emergency medical service; OR, odds ratio; SOFA, Sequential Organ Failure Assessment.

**Figure 2:**
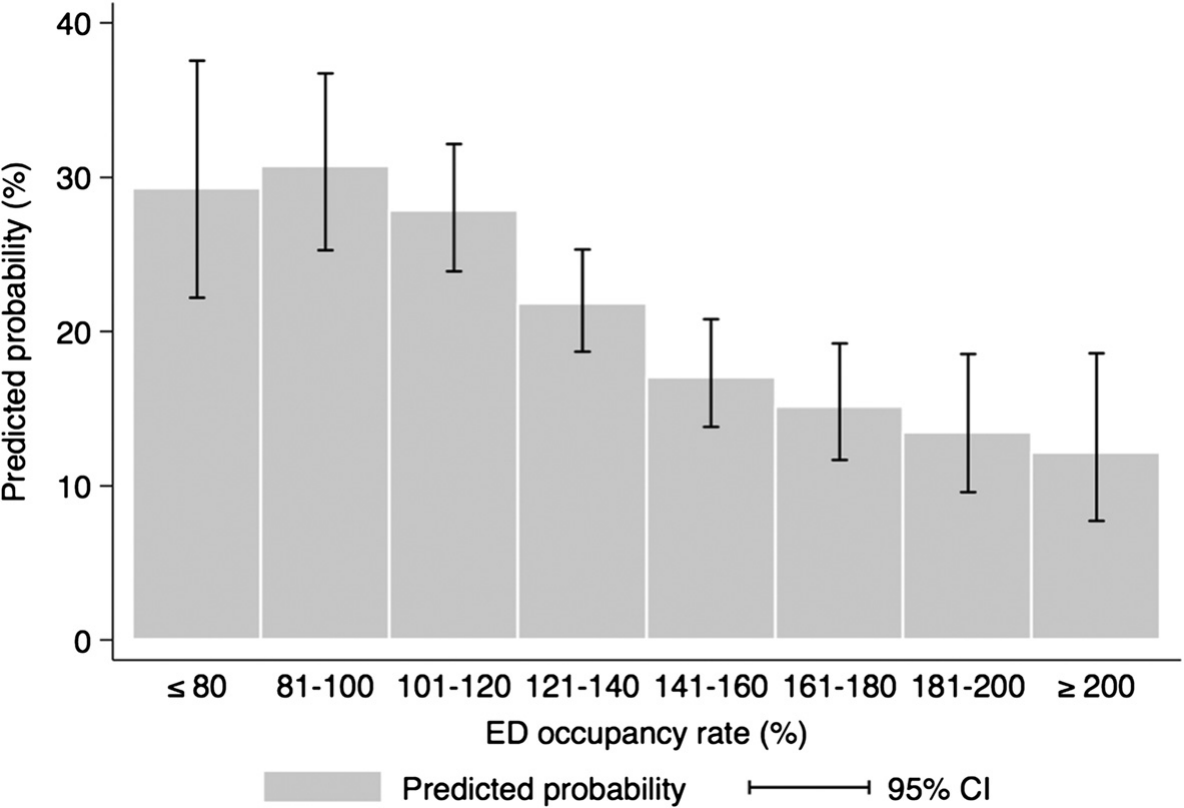
Predicted probabilities of compliance with the resuscitation bundle according to the emergency occupancy rate; CI, confidence interval.

When the ED occupancy rate was included as a continuous variable in the model, instead of the tertiles, it also had a negative correlation with the overall compliance (OR of 10% increase of the ED occupancy rate, 0.90; 95% CI, 0.84 to 0.96, *P* = 0.002). Predicted probabilities of the overall compliance according to the ED occupancy rate are shown in Figure 2.

Additional regression models revealed that the high crowding group or higher ED occupancy rates were significantly associated with decreasing compliance with bundle elements, including early broad-spectrum antibiotics and the achievement of Scvo_2_ ≥70% (Table 4).

**Table 4:** Adjusted odds ratio for the completion of each intervention of the resuscitation bundle

**Variables**	**Adjusted OR (95% CI)**	***P***
Serum lactate measurement		
Intermediate crowding group^a^	0.91 (0.51 to 1.63)	0.747
High crowding group^a^	0.73 (0.38 to 1.41)	0.346
ED occupancy rate (+10%)^b^	0.95 (0.88 to 1.03)	0.206
Blood cultures before antibiotics		
Intermediate crowding group^a^	1.31 (0.52 to 3.30)	0.561
High crowding group^a^	0.80 (0.30 to 2.16)	0.659
ED occupancy rate (+10%)^b^	0.98 (0.86 to 1.10)	0.711
Early broad-spectrum antibiotics		
Intermediate crowding group^a^	0.76 (0.50 to 1.15)	0.193
High crowding group^a^	0.57 (0.35 to 0.91)	0.019
ED occupancy rate (+10%)^b^	0.93 (0.88 to 0.98)	0.018
Intravenous fluid challenge		
Intermediate crowding group^a^	1.20 (0.60 to 2.39)	0.603
High crowding group^a^	0.86 (0.40 to 1.84)	0.697
ED occupancy rate (+10%)^b^	0.99 (0.91 to 1.10)	0.974
CVP ≥8 mmHg achieved		
Intermediate crowding group^a^	1.07 (0.69 to 1.65)	0.772
High crowding group^a^	0.83 (0.50 to 1.37)	0.462
ED occupancy rate (+10%)^b^	0.95 (0.89 to 1.01)	0.095
MAP ≥65 mmHg achieved^c^		
Intermediate crowding group^a^	0.29 (0.06 to 1.45)	0.133
High crowding group^a^	0.34 (0.07 to 1.79)	0.203
ED occupancy rate (+10%)^b^	0.93 (0.80 to 1.10)	0.442
Scvo_2_ ≥70% achieved		
Intermediate crowding group^*a*^	0.83 (0.53 to 1.29)	0.408
High crowding group^a^	0.42 (0.25 to 0.7)	0.001
ED occupancy rate (+10%)^b^	0.90 (0.85 to 0.96)	0.001

^a^Reference category was the low crowding group; ^b^included in models instead of the tertile groups; ^c^Variables with significance of *P* <0.05 were adjusted without mandatory inclusion.

CI, confidence interval; CVP, central venous pressure; ED, emergency department; MAP, mean arterial pressure; OR, odds ratio.

### Length of stay and in-hospital mortality

There was a significant trend toward increasing ED LOS over the tertiles (*P* = 0.002) (Table 5). As for in-hospital stay, the differences or trends were statistically marginal, although there were similar tendencies. In-hospital mortality rates were 16.3% in the low crowding group, 14.4% in the intermediate crowding group, and 18.4% in the high crowding group, respectively (*P* = 0.478). The mortality rate was slightly higher in the high crowding group compared with the other groups combined (low and intermediate crowding group), but these differences were not statistically significant (18.4% versus 15.4%, *P* = 0.288). If we considered only days and evenings on weekdays when ED crowding was more severe, there was a significant difference among the three groups (*P* = 0.019). In particular, the mortality rate of the high crowding group was significantly higher than that in the intermediate crowding group during this time period (*P* = 0.045).

**Table 5:** Length of stay and in-hospital mortality

	**Overall group**	**Low crowding group**	**Intermediate crowding group**	**High crowding group**	***P***
	**(number = 770)**	**(number = 276)**	**(number = 250)**	**(number = 244)**	
ED LOS, hours^a^	20 (8 to 34)	16 (9 to 31)	20 (8 to 36)	24 (9 to 46)^b^	0.009
ICU LOS, days	3 (2 to 6)	3 (2 to 9)	3 (2 to 6)	3 (2 to 6)	0.379
In-hospital LOS, days					
All patients	12 (7 to 22)	11 (7 to 20)	13 (8 to 23)	12 (8 to 22)	0.051
Survivors	12 (8 to 22)	11 (7 to 20)	13 (9 to 22)	13 (8 to 22)	0.048
In-hospital mortality	126 (16.4)	45 (16.3)	36 (14.4)	45 (18.4)	0.478

Data are shown as median with interquartile ranges or number (%). ^a^*P* for trend <0.05 among the tertile groups; ^b^*P* <0.05 compared with the low crowding group after Bonferroni correction. ED, emergency department; ICU, intensive care unit; LOS, length of stay.

## Discussion

Our study showed that ED crowding was associated with poor compliance with the resuscitation bundle, which was based on key SSC guideline elements for management of severe sepsis and septic shock [8]. The achievement of each bundle task was delayed as the level of ED crowding increased. This resulted in a reduction of the completion rate of the entire bundle, which potentially implies a low quality in sepsis care.

To save vital organs and lives, treatment is clearly time-sensitive for patients with severe sepsis and septic shock, as well as acute stroke or acute myocardial infarction [7,18,19]. Primary goals of initial management are early recognition and resuscitation to optimize hemodynamic status within the first few hours, which are represented by the SSC resuscitation bundle. This bundle remains a challenge to perform and has not been widely adopted in practice, although it was associated with performance improvement in sepsis care, and the reported hospital mortality rates were reduced [4,5,9,10,29-31].

Accomplishing the goals of bundle application within the specific time limits requires prompt and effective coordination of hospital resources. ED crowding causes resource shortage and interferes with the ED process in the care of patients with sepsis, which might be the plausible mechanisms linking ED crowding to sepsis care [13,19]. Hence, as our study showed, ED crowding may delay critical ED services during management of severe sepsis and septic shock.

Most of all, it is an important finding that the rate of early antibiotic use was negatively affected by ED crowding since there are number of studies that showed delay in antibiotics affects overall mortality of sepsis patients [4,8,32,33]. Because there is a possibility that an antibiotic delay could be a mechanism by which ED crowding might affect outcomes of patients with sepsis, further studies on this are needed.

We also found several significant factors associated with compliance, such as body temperature, initial presentation of cryptic shock, and the experience of the physician or nurse. The results are consistent with our previous research [21]. In addition to ED crowding, these might be important factors that should be focused on in future interventions.

In a previous study [21], we did not find a significant association between ED crowding and compliance, but it included a smaller number of patients and limited data. We examined overcrowding of the entire ED, and the primary outcome was less strict (adherence to six or seven interventions). We, therefore, performed a multifactorial reanalysis focusing on ED crowding of specific sections and the overall compliance with the entire resuscitation bundle.

To solve the ED crowding problem, multi-factorial, hospital-wide approaches are needed, such as increasing resources and demand management [13]. For instance, a multidisciplinary response team including experienced physicians or nurses and effective hospital bed management could be beneficial. In future studies, we should evaluate interventions to improve compliance with the bundle when crowding is severe.

Early admission to the ICU could be helpful for avoiding ED crowding. However, ED crowding usually occurred when there was a lack of ICU or general ward capacity, and thus the ED input of patients increased. The study hospital has also been experiencing a problem with a shortage of ICU beds as well as ED crowding. Although patients with severe sepsis or septic shock were given priority to the critical care unit in the ED, this practice did not prevent the delay of important interventions.

Reported ED occupancy rates have been diverse, with the median rate being 80 to approximately 110% in some studies [19,26,27]. Little is known about the ED occupancy rate thresholds at which adverse crowding effects occur, and they may vary with each hospital because each ED has different resource structures or capacities, and adverse effects occur when the degree of crowding exceeds these relative capacities. Therefore, although the results cannot be generalized to other EDs, this study showed that crowding could have negative effects on the quality of sepsis care. In addition, the ED calculation of occupancy rate in this study included all patients in the waiting zone. This could be why the ED occupancy rate was higher than the rates reported in other studies.

The patient population with severe sepsis or septic shock might be displaced from receiving care in the dedicated care zone by other critically ill patients. We found that the critical care zone was more often filled to capacity in the high crowding group. When we additionally adjusted for this through multivariate analysis, there was no change in the association between ED crowding and compliance. Even though this was not a significant factor in this study, further investigation is required regarding whether too many critically ill patients in an ED affect the quality of sepsis care regardless of ED crowding.

Our study has several limitations as a single-center, retrospective, observational study. First, there might be effects of unobserved bias that we were unable to fully control, and data collection was partially dependent on the accuracy of documentation in the medical records. Second, the results may not be readily applicable to other institutions that have different settings and should be cautiously interpreted. For hospitals with low crowding in the ED, our results cannot be directly applied. Third, the effect of ED crowding was not evaluated by using other methods. However, the ED occupancy rate showed a correlation with another validated scale reflecting the number of physicians, and it was useful to predict adverse outcomes despite its simplicity [26]. Fourth, there are dimensions of sepsis care compliance that we cannot quantify although we have broken down care to the elements of the bundle. The benefit of the bundle is probably the overall combination of interventions. Fifth, the finding that overcrowding influences patient treatments is not novel, and this is not an intervention study regarding raising compliance in cases of overcrowding. However, considering the clinical significance of the sepsis care bundle, and even standing alone, completion of the sepsis care bundle is considered to be a difficult treatment option to achieve according to several studies including recently published articles, this study has a value in that it shows an association between ED crowding and, specifically, the sepsis care bundle completion [8,9,31].

## Conclusions

ED crowding had adverse effects on compliance with the resuscitation bundle in the management of severe sepsis or septic shock and was significantly associated with lower compliance with the entire resuscitation bundle. ED crowding was also associated with decreased likelihood of the timely implementation of the bundle elements, including early broad-spectrum antibiotic administration and the achievement of Scvo_2_ ≥70%. Severe ED crowding might be associated with increasing in-hospital mortality.

## Key messages

• ED crowding was significantly associated with lower compliance with the entire resuscitation bundle in the management of severe sepsis or septic shock.

• ED crowding was also associated with a decreased likelihood of the timely implementation of the bundle elements.

## Abbreviations

BP: Blood pressure; CI: Confidence interval; CVP: Central venous pressure; DNAR: Do not attempt resuscitation; ED: Emergency department; EGDT: Early goal-directed therapy; LOS: Length of stay; MAP: Mean arterial pressure; OR: Odds ratio; Scvo2: Central venous oxygen saturation; SOFA: Sequential organ failure assessment; SSC: Surviving sepsis campaign.

## Competing interests

The authors declare that they have no competing interests.

## Authors’ contributions

TGS collected and analyzed the data and drafted this manuscript. IJJ conceived and designed the study, analyzed the data, and wrote the final manuscript. DJC collected data and assisted with analyzing the data and drafting the manuscript. MJK collected data and assisted with analyzing the data and drafting the manuscript. KJ assisted with analyzing the data and drafting the manuscript. GYS assisted with analyzing the data and drafting the manuscript. MSS collected data and assisted with analyzing the data and drafting the manuscript. SYL assisted with analyzing the data and drafting the manuscript. KJS contributed to analysis and interpretation of the data. YKJ contributed to analysis and interpretation of the data. All authors have read and approved the final manuscript.
